# Extreme Mercurial Salivation

**Published:** 1845-11

**Authors:** A. H. Peck

**Affiliations:** Port Gibson


*Extreme Mercurial Salivation.*—To the editor of the Boston Medical and Surgical Journal:—Dear Sir, As the subject of mortification of the mouth appears to be attracting the attention of several writers in your late Nos., and a doubt expressed as to the cause of it, whether attributable to mercury or not, I will offer a few remarks. I do this the more readily when considering the fatality of the disease ordinarily.

Here it is not only a popular opinion, but one sanctioned by the medical public, that mortification of the mouth following fever after the use of mercury, is as much the extreme grade of salivation as is the simplest ptyalism produced by that agent. It is termed dry salivation. It has the mercurial odor, and it yields to the same remedies, medicated, however, proportionally to the increase of violence. In your No. 25, Vol. IV., Aug. 2, 1831, you did me the honor of republishing two cases reported by me to the Transylvania Journal of Medicine. They were children of 8 and 11 years. They had been very stubborn fever cases previous to the appearance of the gangrene of the mouth. I cut away portions of it, and freely insinuated a strong lotion of muriatic acid and water, diluting it as the disease appeared yielding. The accompanying fever was kept down by active doses of the comp. pow. jal. In a few days they were relieved, notwithstanding in one of them half the inside of the upper jaw and cheek adjoining was thus diseased, with all the accompanying symptoms of hideously swollen face, &c., &c. Since that time I have had cases of all ages, from infancy to the octogenarian, and of all grades, from the mildest increase of saliva to mortification, and find the remedy equally adapted to all. I will give some particulars of a case in point.

November 16th, 1831, I was called to Mr. P. B., one of the companions of Daniel Boon, a very old man. He had an attack of congestive fever, and was treated successfully by Dr. S. A few days after its disappearance, mortification of the mouth ensued. The common remedies were used in vain, and the disease extended rapidly. I found the entire inside of his mouth covered with a soft brownish mortification, with an intolerable stench; he was prostrated, and in a comatose state. I removed portions of the disease, and then applied a lotion of equal portions of muriatic acid and water to the parts freely. This was persevered in several times a day, for several days. His bowels were kept open. His disease was removed in three days.

The only fatal case I have to relate, was a child two years of age. It was in the autumn of 1833. Her disease had been an obstinate diarrhœa, and it was not arrested when the gangrene supervened. She had just changed climates, too, and a cholera atmosphere had been and might still be said to be prevailing. She was a thousand miles north of home.

In all the other cases, the disease for which the mercurial preparation—the proto-ch. hydrarg.—had been given, had yielded *before* the mortification appeared; an important consideration, probably, in the prognosis. The disease is less often met with now than formerly; indeed, some years it is more frequently met with than others. Several years after the cases alluded to were reported, I observed, in the medical journals of the day, muriatic acid mentioned as the favorite remedy of M. Velpeau in the treatment of mercurial salivation.

*Port Gibson, Aug.* 26, 1845. A. H. PECK.

